# Torch Oscillation-Induced Microstructure Evolution, Deformation Mitigation and Mechanical Property Enhancement in Thin-Plate 5083 Aluminum Alloy Butt Welds

**DOI:** 10.3390/ma19204344

**Published:** 2026-10-15

**Authors:** Yuwen Wang, Yiming Chen, Xue Li, Jie Liu, Qing Xue, Zhenhua Wang, Hairui Wang, Jie Xu, Yu Fan, Lin Wang, Zheng Chen

**Affiliations:** 1 School of Materials Science and Physics, China University of Mining and Technology, Xuzhou 221116, China; yuwen.wang@cumt.edu.cn (Y.W.); 13990230460@163.com (Y.C.); j.xu@cumt.edu.cn (J.X.); fanyu@cumt.edu.cn (Y.F.); wanglin1990@cumt.edu.cn (L.W.); 2 School of Materials Science and Engineering, Institute of Materials Joining, Shandong University, Jinan 250061, China; 3 China Special Equipment Inspection & Research Institute, Beijing 100029, China

**Keywords:** 5083 aluminum alloy, cold metal transfer welding, torch oscillation, microstructure, deformation, mechanical properties

## Abstract

AA5083 Al-Mg alloy is widely applied in lightweight marine and transportation structures, yet thin-plate arc welding suffers from prominent out-of-plane deformation and coarse columnar-grain issues originating from concentrated arc heat input. This work adopted sinusoidal torch-oscillation-assisted CMT to conduct butt-welding experiments on 2 mm-thick AA5083-H112 sheets with ER5183 filler wire. Five oscillation amplitudes ranging from 0 mm to 4 mm were set to explore the coupled effects of oscillation amplitude on weld geometric features, three-dimensional base-plate deformation, weld-zone microstructural evolution, crystallographic texture and tensile-fracture behaviors. The results reveal that appropriate torch oscillation redistributes arc heat flux and introduces molten-pool stirring. It mitigates thin-plate out-of-plane deformation, refines grains, and promotes equiaxed-grain formation with a slight decrease in the measured texture intensity. The joint’s comprehensive mechanical performance is highest at an oscillation amplitude of 2 mm among the tested conditions in this work: ultimate tensile strength, yield strength, and elongation attain 219.03 MPa, 112.14 MPa, and 18.45%, accompanied by typical ductile-dimple fracture features. Nevertheless, amplitudes exceeding 3 mm destabilize the molten pool and trigger pores and local lack-of-fusion defects, leading to sharp performance deterioration and brittle fracture. The competitive relationship among deformation relief, grain refinement, and defect generation determines the valid amplitude window. This study provides an experimental reference for high-quality CMT welding of thin-gauge 5083 aluminum alloy via torch-oscillation process regulation.

## 1. Introduction

AA5083 Al-Mg alloy is widely applied in lightweight marine and transportation structures, including ship hulls and superstructures, high-speed train body panels, and automotive lightweight components, owing to its high specific strength, good corrosion resistance, and weldability [1,2,3]. In these applications, thin AA5083 sheets are usually joined into large-area panels, where welding-induced out-of-plane deformation affects panel fit-up and assembly accuracy, while coarse columnar grains and defects such as porosity and lack of fusion reduce joint ductility and load-bearing capacity [4,5]. Thin-sheet AA5083 aluminum alloy joints require control of welding deformation, microstructure, and defects to retain their load-bearing capacity. The alloy’s use in marine and transportation structures has motivated studies of gas tungsten arc welding (GTAW) and gas metal arc welding (GMAW), including comparisons with other structural aluminum alloys [1,2,3]. These studies link the mechanical response of welded joints to differences between the fusion zone, heat-affected zone, and base material. Even within AA5083, plate thickness changes the metallurgical and mechanical response to GMAW [4], while hydrogen in ER5183 filler wire affects porosity, tensile strength, and fracture behavior [5]. Comparable thickness- and filler-dependent effects have also been reported for other Al-Mg and Al-Mg-Si alloy systems, where fusion-zone grain structure, heat-affected-zone softening, and defect population jointly determine the tensile and fatigue response [2,3]. Process selection therefore requires consideration of both the solidified weld structure and the defects that interrupt load transfer.

Thin-sheet welding adds a geometric constraint: sufficient through-thickness fusion must coexist with limited out-of-plane deformation. Studies of AA5083 using static thermal tensioning and heat sinks show that thermal management affects distortion, residual stress, and fatigue behavior [6,7]. Recent numerical work on thin-sheet AA6082-T6 joints further relates residual stress to welding conditions [8], while fatigue assessment of AA5059 explicitly accounts for welding deformation and residual stress [9]. Coupled simulations of thin AA6061-T6 welded sheets likewise emphasize the combined roles of joint geometry, local material properties, and residual stress in mechanical response [10]. Taken together, these studies indicate that dimensional quality and mechanical performance are closely coupled in thin-sheet joints, so that reducing distortion without maintaining adequate penetration or defect control may not improve the overall joint reliability. For thin-sheet butt joints, evaluating dimensional quality alongside strength and ductility is therefore more informative than judging quality from a single indicator.

Cold metal transfer (CMT) provides a means of limiting heat input through coordinated electrical control and wire retraction during short-circuit transfer. Foundational studies established its applicability to thin aluminum sheets and examined its penetration characteristics [11,12]. Subsequent investigations of AA5083/AA6082, 2A14, and 2A12 joints showed that CMT welding conditions influence microstructure and mechanical properties [13,14]. Recent comparisons with conventional GMAW and studies of dissimilar aluminum joints reinforce the dependence of CMT weld quality on process selection and parameters [15,16]. For AA5083 specifically, changing the Ar–He shielding-gas composition altered weld formation, porosity, microstructure, and tensile performance [17]. Related CMT-based deposition studies identify arc mode and filler-wire batch as additional factors affecting porosity [18,19]. These deposition studies further suggest that the spatial and temporal distribution of arc heat, rather than the average heat input alone, plays a decisive role in pore formation and grain structure. Thus, low heat input is one component of quality control; the distribution of arc action and the conditions governing defect formation also warrant attention.

Torch oscillation redistributes arc action across the welding direction and changes the conditions for molten-pool transport and solidification. Experimental and numerical studies of swing-arc narrow-gap GMAW connect the spatial action of the arc with heat transfer, fluid flow, and defect formation [20,21,22]. In tandem narrow-gap welding of AA5083, arc interaction and interwire angle also influence lack of fusion, porosity, and bubble escape [23,24]. These results establish that the arrangement and movement of the heat source affect weld soundness. More recently, a comparison of GTAW and SpinArc-GMAW examined the microstructure, hardness, and corrosion behavior of AA5083 bead-on-plate welds [25]. Collectively, these studies demonstrate that oscillating or weaving the arc can homogenize the thermal field and improve sidewall fusion in narrow-gap and deposition configurations. However, narrow-gap sidewall fusion and bead-on-plate processing impose different geometric constraints from single-pass thin-sheet butt welding. The effect of increasing lateral oscillation on root penetration therefore requires evaluation in the actual sheet-joint configuration.

Torch oscillation-induced grain refinement depends on both the alloy and the imposed thermal and fluid-flow conditions. Classical work on AA5052 established a connection between arc oscillation, grain structure, and solidification cracking [26], while a recent review places oscillation within the broader range of aluminum weld-metal refinement methods [27]. Experiments on alternating Ar–He supply in 5A06 TIG welding and ultrasonic-assisted AA5083 TIG welding provide further examples of grain refinement through modified arc or pool conditions [28,29]. Other approaches, such as external magnetic fields, pulsed arc, and beam oscillation, have also been explored to refine weld grains and suppress solidification defects in aluminum alloys, confirming that forced disturbance of the molten pool is an effective refinement route [27,28,29]. The underlying mechanisms require careful distinction. Experiments on magnesium welds associated transverse arc oscillation with dendrite fragmentation through local reheating and remelting [30]; this result provides a mechanistic reference rather than direct evidence for AA5083 CMT welding. Moreover, arc-oscillation experiments produced pronounced refinement in AA5052 but limited refinement in AA6061 [31]. These differences support examining grain structure and mechanical performance separately before attributing a strength improvement to refinement.

Recent aluminum wire-arc additive manufacturing (WAAM) studies show how thermal conditions, oscillation, and defects interact during CMT processing. Work on AA2319 demonstrates that nucleant additions and CMT heat-input conditions jointly influence the resulting grain structure [32]. Wei et al. found that different oscillation paths affected porosity, grain size, and tensile behavior in CMT-WAAM AA2319 [33]. Torch oscillation also improved interfacial behavior in deposited 2319/5356 dissimilar alloys [34]. Particularly relevant to amplitude selection, a 2026 study of AA4043 reported the best balance of mechanical properties at an intermediate weaving width, although the largest width produced the finest grains [35]. These additive-manufacturing studies consistently show that increasing the oscillation or weaving width does not monotonically improve joint performance, because grain refinement and defect formation are affected simultaneously by the oscillation parameters. Together, these findings motivate concurrent assessment of refinement and defects. Their repeated deposition cycles and build constraints nevertheless differ from the thermal history and root-fusion requirement of single-pass sheet welding.

For 2-mm AA5083 CMT butt joints, the question is how increasing torch oscillation amplitude balances grain refinement and surface flatness against penetration and tensile ductility. The reviewed narrow-gap studies establish links between heat-source configuration and defect formation [20,23,24], while recent deposition studies show that the smallest grains need not yield the best mechanical response [33,35]. Nevertheless, systematic amplitude-dependent data for single-pass thin-sheet CMT butt welding remain limited, and the coupled effects of oscillation amplitude on weld geometry, three-dimensional deformation, crystallographic texture, and tensile fracture behavior have not been clarified for 2-mm AA5083-H112 joints. An assessment that combines weld geometry, grain structure, regional surface topography, and tensile behavior can resolve whether an apparent improvement in one quality measure conceals deterioration in another. This distinction is necessary for selecting a favorable amplitude under defined welding conditions.

Here, we investigate sinusoidal torch-oscillation CMT butt welding of 2-mm AA5083-H112 sheets with ER5183 filler wire. We compare amplitudes of 0, 1, 2, 3, and 4 mm, using a fixed oscillation frequency of 5 Hz for the oscillated conditions and unchanged nominal settings for the remaining welding parameters. Weld-surface and cross-sectional observations characterize formation and penetration, while three-dimensional topography measurements quantify surface height variation over a defined plate region. Electron backscatter diffraction characterizes grain size, morphology, and texture; tensile testing and fractography assess strength, ductility, and fracture behavior. Among the tested conditions, the 2-mm amplitude provides the best tensile performance within the present experimental parameter set. Further increases continue to refine the grains and reduce surface height variation, but joint performance deteriorates alongside penetration loss and porosity. The study identifies the resulting trade-off between geometric quality, grain refinement, and defect formation when selecting an oscillation amplitude for thin-sheet CMT welding.

## 2. Materials and Methods

### 2.1. Materials and Welding Process

In this work, AA5083-H112 aluminum alloy plates serve as the base plates for butt welding. The plate has a length of 200 mm, a width of 60 mm, and a thickness of 2 mm. The filler wire was ER5183 (Al-Mg-Mn alloy) with a diameter of 1.2 mm. The chemical compositions of the base plate and filler wire are listed in Table 1. The 5183 aluminum alloy filler wire was supplied by Safra S.p.A., Brescia, Italy, and the 5083-H112 aluminum alloy base plates were provided by Shanghai Dongxu Metal Products Co., Ltd, Shanghai, P.R. China.

**Figure 1:**
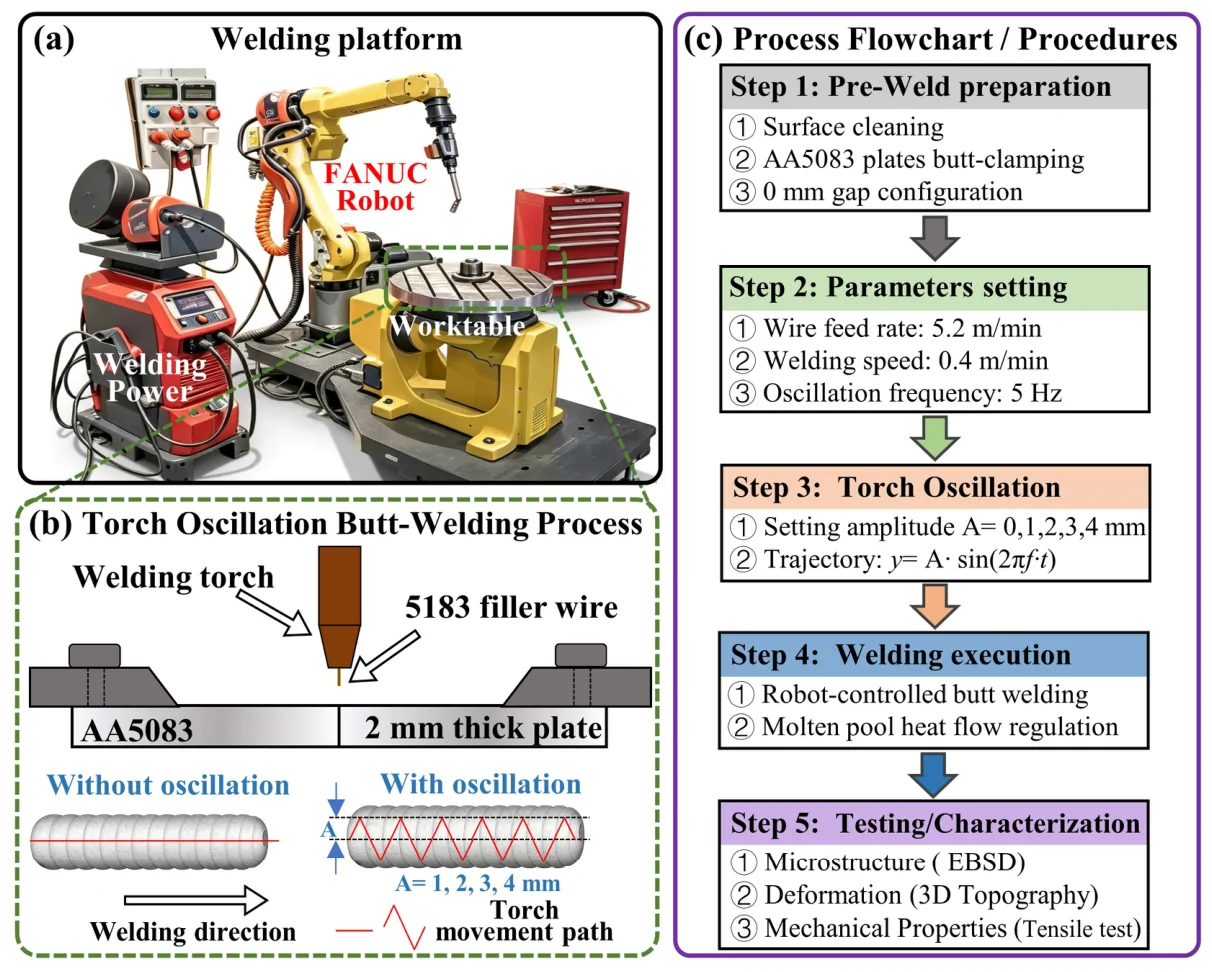
Welding platform, schematic of the butt-welding process, and step-by-step welding procedures: (**a**) photograph of the robotic CMT welding platform; (**b**) schematic of the torch-oscillation butt-welding configuration and the sinusoidal oscillation trajectory at amplitudes of A = 1–4 mm; (**c**) flowchart of the welding procedures from pre-weld preparation to testing and characterization.

The welding platform is shown in Figure 1a. It mainly consisted of a TPS 500i CMT welding machine (Fronius International GmbH, Wels, Austria), a FANUC M-20iA industrial robot (FANUC Corporation, Yamanashi, Japan), a WF 25i REEL wire feeder (Fronius International GmbH, Wels, Austria), a welding workbench (80 cm in diameter), and a robot teach pendant. The schematic of the butt-welding configuration is shown in Figure 1b, and the step-by-step welding procedure is summarized in Figure 1c. Before welding, the base plates were cleaned using anhydrous ethanol. After natural drying, they were fixed on the experimental platform using fixtures. No groove was prepared on the base plates, and the assembly gap was 0 mm. The CMT mode was selected for the butt welding experiments. The wire feed speed was uniformly set to 5.2 m/min, and the welding speed was 0.4 m/min. The specific process parameters are listed in Table 2. As shown in Figure 1b, the torch oscillation trajectory followed a sinusoidal path. The maximum deviation from the central axis was defined as the oscillation amplitude (A). Five groups of comparative experiments were designed: no torch oscillation, i.e., an A value of 0 mm, and torch oscillation amplitudes of 1–4 mm. Through sinusoidal oscillation of the torch, the heat flow behavior of the molten pool was regulated, thereby improving weld formation, optimizing microstructure, and enhancing performance. High-purity argon was used as the shielding gas at a flow rate of 25 L/min, and the nozzle height was set to 15 mm.

The nominal heat input was calculated as Q = ηUI/v, where U is the arc voltage, I is the welding current, and v is the welding speed. The oscillation amplitude showed no obvious influence on arc voltage and welding current. With U = 12.2 V, I = 70 A, and v = 0.4 m/min (6.667 mm/s), the nominal heat input was approximately 102.48 J/mm and did not vary appreciably among the five amplitudes (A = 0–4 mm). Torch oscillation did not change the total nominal heat input but redistributed the arc heat spatially, reducing the peak heat flux density and the through-thickness heat flux as the amplitude increased.

### 2.2. Microstructure Characterization, Mechanical Test, and Deformation Measurement Test

**Figure 2:**
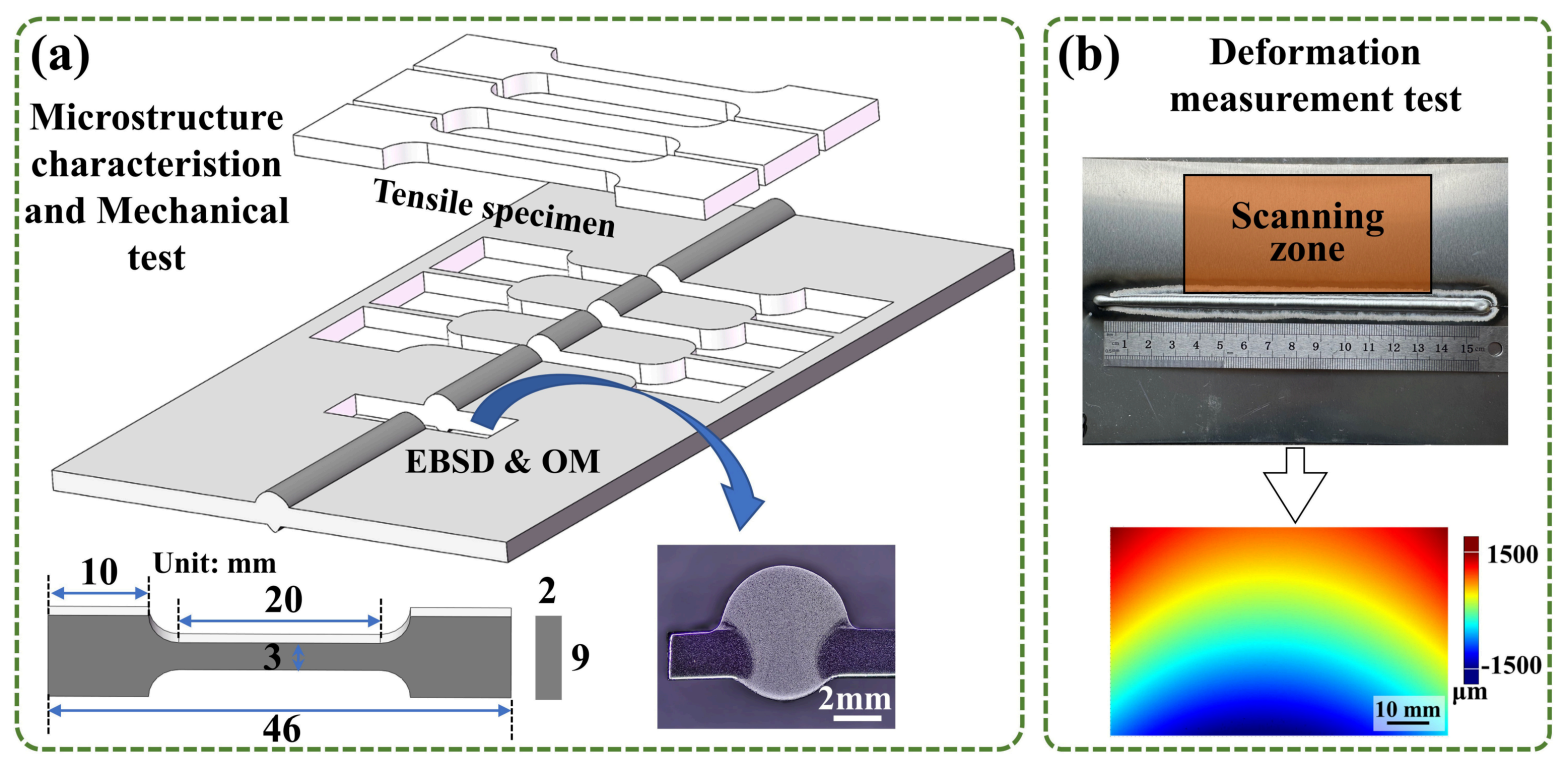
Schematic diagrams of microstructure characterization, mechanical test, and deformation measurement test: (**a**) microstructure characterization and mechanical test, (**b**) deformation measurement test.

After welding, specimens were cut using a wire electrical discharge machine (Model DK7735, Shandong Guanke CNC Equipment Co., Ltd., Jining, Shandong, China). The macrostructure and microstructure of the butt welds were analyzed using an optical microscope (OM) and electron backscatter diffraction (EBSD), as shown in Figure 2a. Specimens were ground with 120–800 grit sandpaper, then polished to a mirror finish. After grinding and polishing, the specimens were electrolytically etched in a fluoroboric acid aqueous solution (HBF_4_, 2.5 vol.%) at room temperature (25 °C) with an applied voltage of 20 V and a current of 300 mA for 60–90 s, and the weld macrostructure was then observed with an optical microscope (Nikon Eclipse LV150N, Nikon Corporation, Tokyo, Japan). EBSD specimens underwent the same grinding and polishing steps, followed by electrolytic polishing to remove the surface residual stress layer. EBSD data were acquired using an Oxford HKL NordlysMax detector (Oxford Instruments, Abingdon-on-Thames, UK) on a JSM-7800F field emission scanning electron microscope (JEOL Ltd., Tokyo, Japan) and processed with ATEX (v3.25) and Channel 5 (v5.1). EBSD mapping was performed with a step size of 5 μm. Each mapped area covered approximately 4000 μm × 1800 μm. A grain was defined when the misorientation between adjacent pixels exceeded 15°. For each oscillation-amplitude condition, two independent regions in the vicinity of the weld center–fusion line were analyzed for statistical reliability.

To evaluate the tensile properties of the butt joints, tensile specimens were cut with the weld as the center, and all specimens were taken from the middle-to-rear section of the weld, where the welding process had reached a relatively stable state. The sampling position was kept consistent for all five conditions. Since the specimen dimensions required by GB/T 228-2002 [36] are too large for the base plate size, non-standard specimens scaled down proportionally were used in this study. The specimen dimensions and sampling locations are shown in Figure 2a. Before testing, the specimen surfaces were ground to reduce the influence of cutting marks. Tensile tests were performed at room temperature using a Mechanical Testing & Simulation (MTS) universal testing machine (Model E45, MTS Industrial Systems (China) Co., Ltd., Shenzhen, China) at a crosshead speed of 1 mm/min. An extensometer was attached to the gauge section to ensure elongation accuracy, and each parameter was tested at least three times.

Meanwhile, the influence of torch oscillation on the surface deformation of the base plates was further evaluated by a laser spectral confocal microscope (Model KC-X1000, KathMatic Technology Co., Ltd., Nanjing, China), as described in Figure 2b. The scanning parameters were set as follows: scanning area of 50,000 μm × 80,000 μm, scanning interval of 250 μm, acquisition frequency of 4500 Hz, and scanning speed of 30,000 μm/s. The acquired forming accuracy data included surface roughness (*S_a_*) and maximum surface height difference (*S_z_*). In this study, these parameters are used to characterize the degree of macroscopic surface waviness and out-of-plane deformation of the base plate over the scanned region, rather than the local micro-scale roughness of the weld bead.

## 3. Results and Discussion

### 3.1. Macroscopic Features and Cross-Sectional Geometry

**Figure 3:**
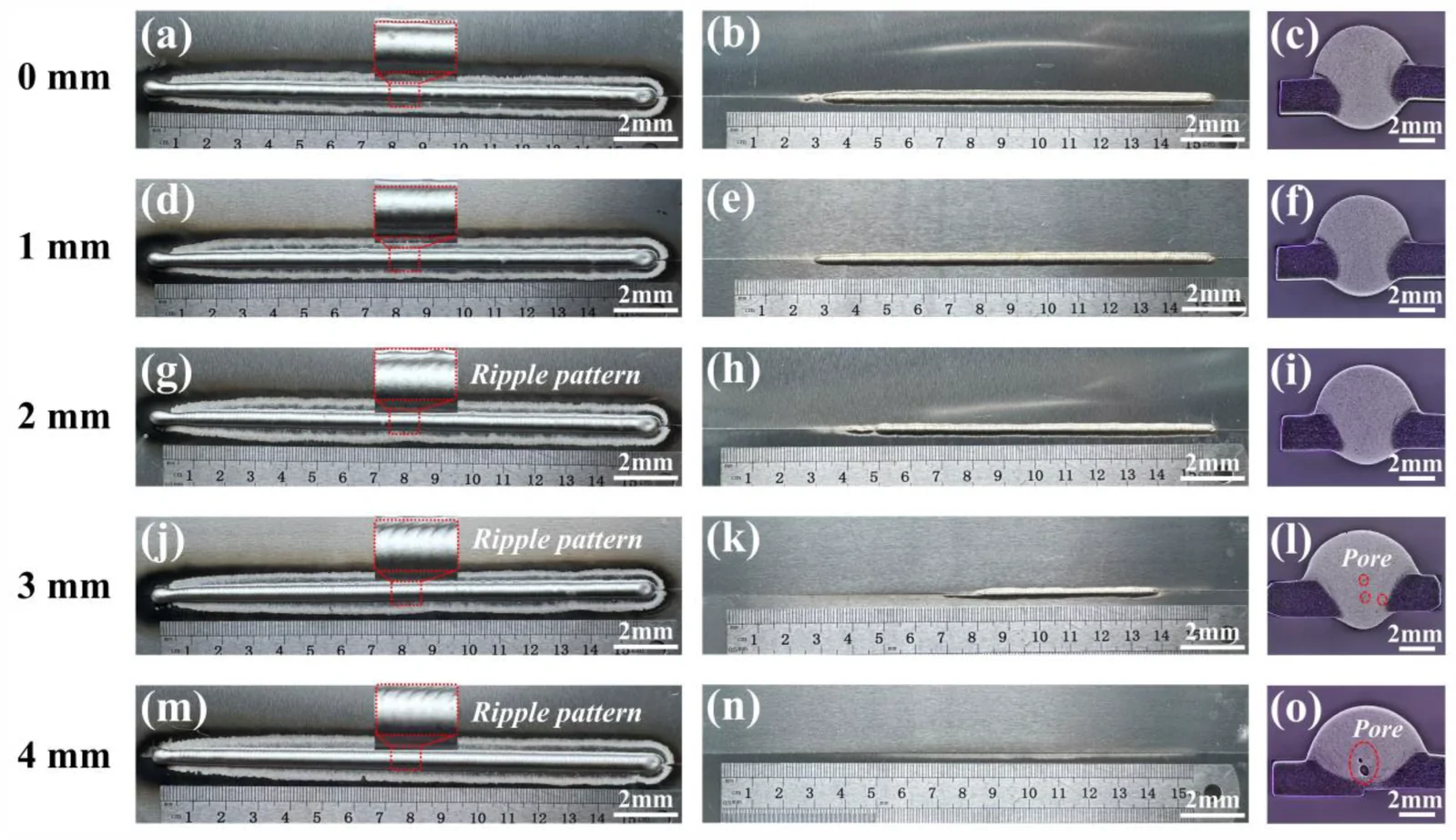
Macroscopic morphology diagram of the butt weld under different process parameters: (**a**–**c**) 0 mm, (**d**–**f**) 1 mm, (**g**–**i**) 2 mm, (**j**–**l**) 3 mm, (**m**–**o**) 4 mm. The red dashed box denotes the locally enlarged view of the stable weld region.

Figure 3 shows the weld macrostructure under no torch oscillation and different oscillation amplitudes. As shown in Figure 3a,d,g,j,m, the top surfaces of all butt welds were continuous and uniform, with no humping or discontinuity, and the differences between them were not obvious. However, the enlarged local regions marked by red dashed boxes reveal clearer differences in weld surface morphology. When the torch oscillation was not employed, the weld surface was smooth and free of ripples. At an A value of 1 mm, faint and intermittent ripples appeared on the weld surface, as described in Figure 3d. When the A value increased to 2 mm, the ripples became more distinct, and they became increasingly pronounced with further increases in amplitude, indicating that torch oscillation significantly promoted ripple formation. Figure 3b,e,h,k,n show the back surfaces of the butt welds under different conditions. Almost the entire weld was fully penetrated without torch oscillation, and a penetration length of about 12.5 cm was formed. As the A value increased, the penetration length gradually decreased, dropping to about 6 cm at an A value of 3 mm. When the A value increased to 4 mm, the weld back surface was completely unpenetrated, indicating that torch oscillation enlarged the molten pool and dispersed the molten heat, effectively alleviating excessive heat concentration. As shown in Figure 3c,f,i,l,o, under the no-oscillation condition, obvious deformation and misalignment occurred on both sides of the weld. After applying torch oscillation, no significant deformation or misalignment was observed in the four groups. However, at an A value of 3 mm, fine pores began to appear, and at an A value of 4 mm, excessive heat dispersion caused the molten pool to solidify too rapidly, preventing pores at the bottom from escaping and resulting in large circular pores.

**Figure 4:**
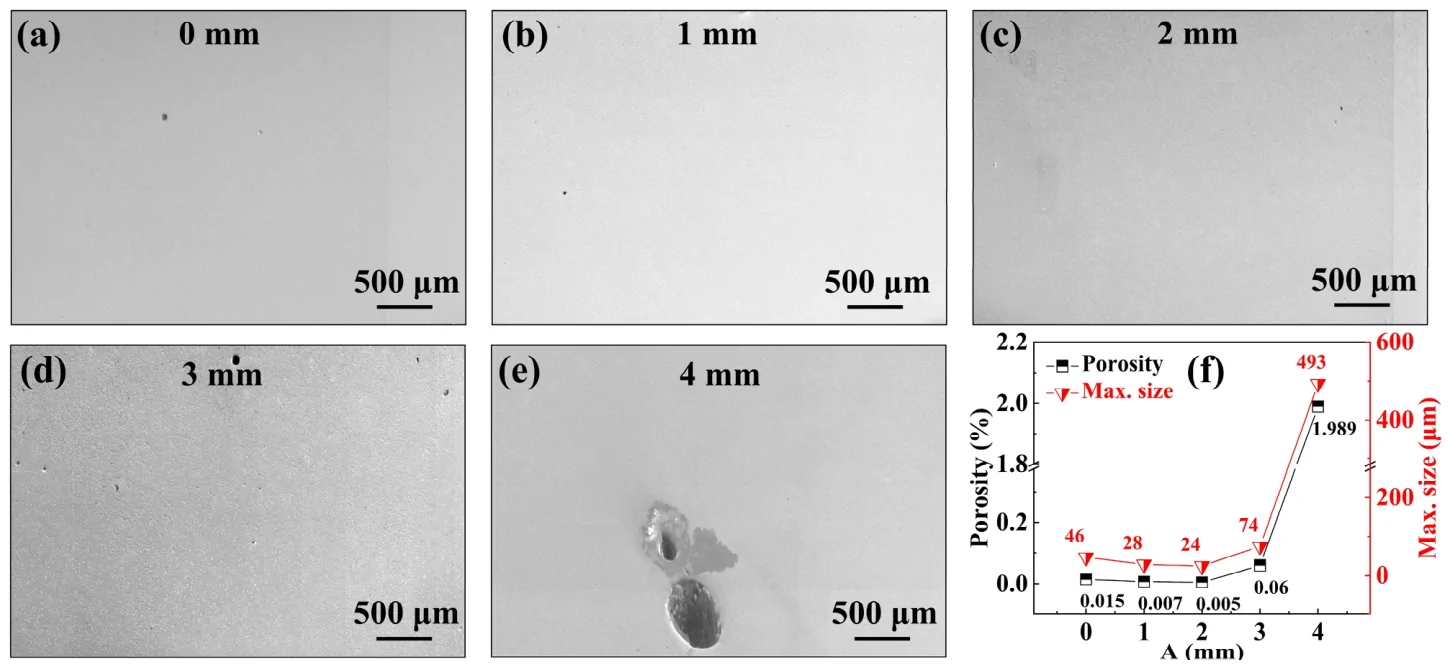
Pore evolution and quantitative statistics of the welds under different oscillation amplitudes: (**a**–**e**) Microstructure and pore morphology of the weld zone at A = 0, 1, 2, 3, and 4 mm; (**f**) Variations of porosity and maximum pore size with oscillation amplitude.

To quantify the defect evolution with oscillation amplitude, the variations in porosity and maximum pore size with oscillation amplitude were measured from cross-sectional micrographs, as shown in Figure 4. The porosity remained below 0.02% at A = 0–2 mm, increased to 0.06% at A = 3 mm, and reached 1.989% at A = 4 mm. Correspondingly, the maximum pore diameter increased from 24 μm to 493 μm. The pores at A = 4 mm were predominantly large and circular and appeared near the weld root, indicating that excessive oscillation destabilized the molten pool and promoted gas entrapment.

**Figure 5:**
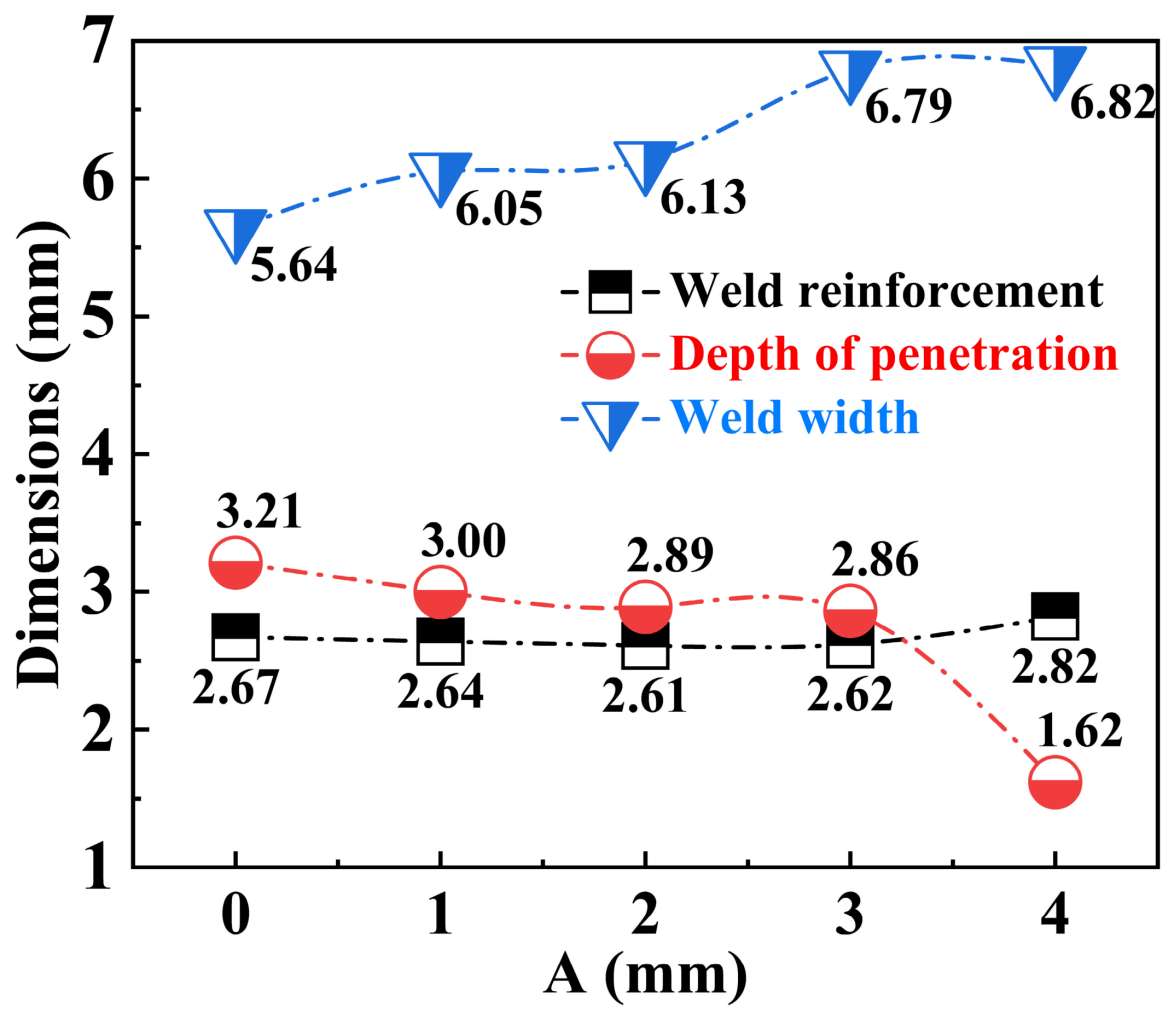
Geometric dimensions of butt weld under different process parameters.

Figure 5 presents the cross-sectional geometry of the welds obtained with and without torch oscillation. The results show that torch oscillation affected the depth of penetration and weld width more significantly than weld reinforcement.

When torch oscillation was not applied, the arc heat source was concentrated on the weld centerline, and a relatively high proportion of heat was conducted along the plate thickness, resulting in limited transverse spreading. When torch oscillation was applied, the arc moved back and forth transversely, dispersing the concentrated heat source in the width direction. This increased the weld width, which continued to increase with oscillation amplitude and gradually stabilized at about 6.8 mm beyond an amplitude of 3 mm, representing an increase of 17.3% compared with the non-oscillated condition.

The penetration depth measured from the cross-sectional macrographs decreases gradually from 3.21 mm at A = 0 mm to 2.86 mm at A = 3 mm, and then drops sharply to 1.62 mm at A = 4 mm. Because the plate thickness is 2 mm, the penetration depth at A = 4 mm is insufficient to fuse through the plate thickness, confirming the complete lack of penetration observed on the back surface in Figure 3n. No lack of penetration is detected at A = 0–3 mm.

As the oscillation amplitude increased, the depth of penetration exhibited a two-stage evolution: a slow decrease followed by a sharp drop. This nonlinear response could be attributed to a critical threshold in the dilution effect of oscillation amplitude on arc heat flux density. At small amplitudes, the concentrated heat was only mildly spread transversely, and the downward heat flux remained sufficient to sustain the advancement of the molten pool bottom. Once the amplitude exceeded the critical threshold, the energy was excessively diluted, and the heat input along the plate thickness became insufficient to support continued expansion of the pool bottom, causing the depth of penetration to shift from gradual shrinkage to a sharp decline.

The weld reinforcement changed little with increasing A values and increased only slightly at an A value of 4 mm. The wire feed speed remained constant, so the total amount of filler metal was essentially unchanged. The oscillation widened the molten pool and spread the filler metal over a wider area, thereby reducing the weld reinforcement. At the same time, the reduced depth of penetration decreased the volume occupied by the pool bottom, and the same amount of filler metal accumulated in a shallower pool, which increased the reinforcement height. These two effects offset each other to some extent, making the weld reinforcement change insignificant.

### 3.2. Surface Morphology and Deformation Characteristics

**Figure 6:**
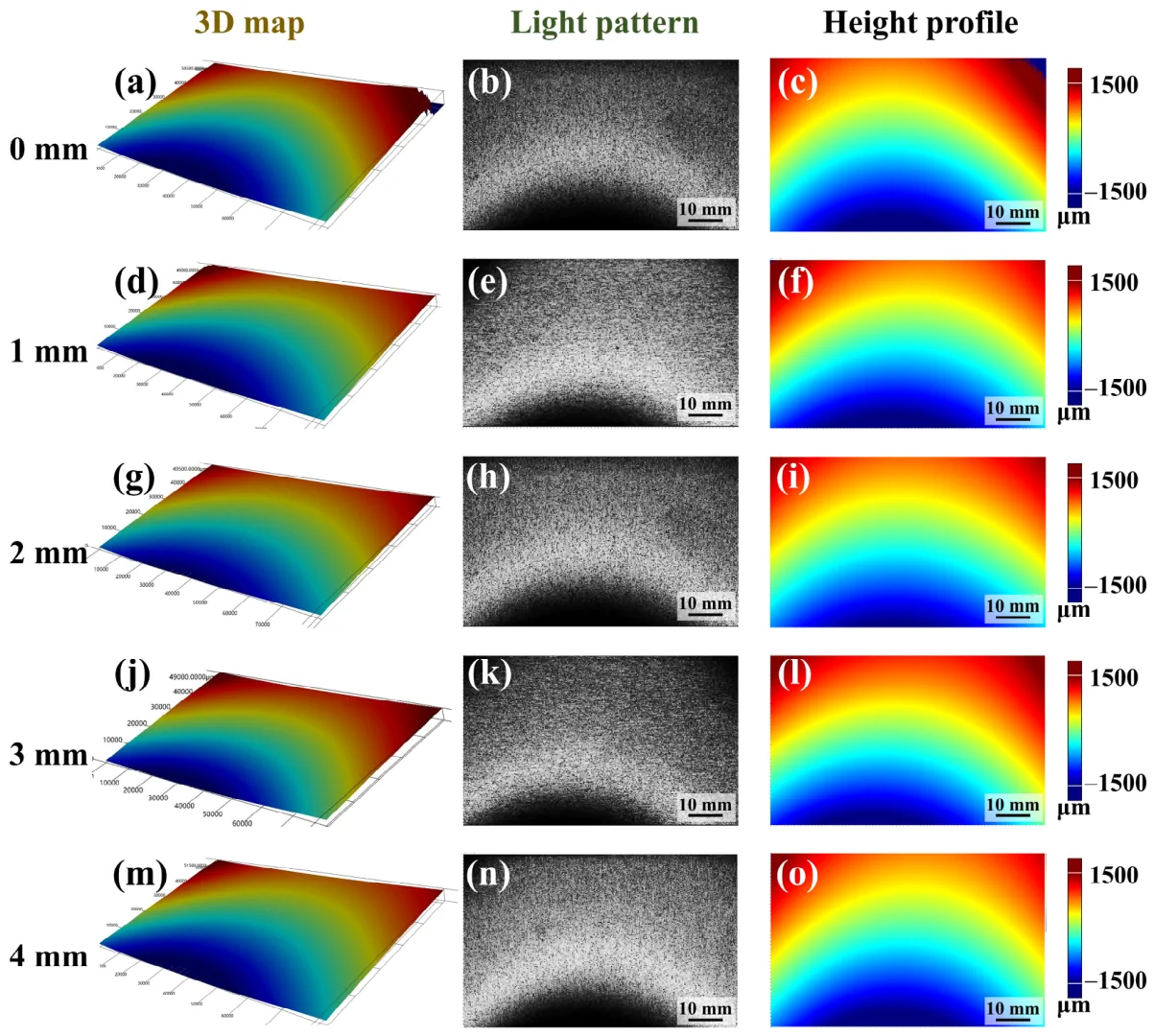
Three-dimensional topography and contour nephograms of base plates fabricated at A values ranging from 0 to 4 mm: (**a**–**c**) 0 mm, (**d**–**f**) 1 mm, (**g**–**i**) 2 mm, (**j**–**l**) 3 mm, (**m**–**o**) 4 mm.

**Figure 7:**
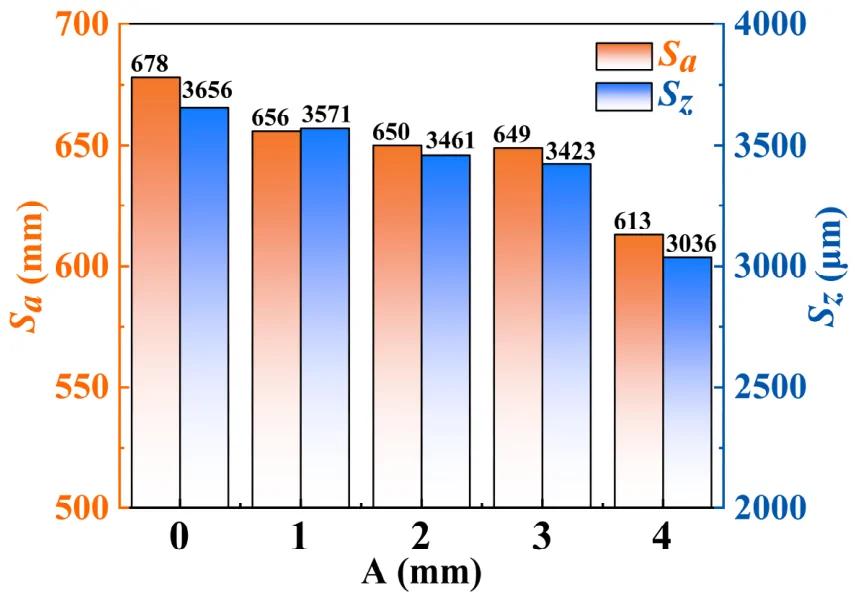
Deformation measurement indicators of base plates fabricated at A values ranging from 0 to 4 mm.

Figure 6 and Figure 7 present the 3D surface morphology and statistical results of the base plates after welding with and without torch oscillation. The color scale represents the surface height distribution, where warm colors indicate protruding regions and cold colors indicate recessed regions. When torch oscillation was not applied (Figure 6a–c), the base plate exhibited obvious out-of-plane deformation, and large height fluctuations occurred on both sides of the weld. The maximum *S_z_* reached 3656 μm, and the surface roughness *S_a_* was 678 μm. Under the torch oscillation condition, the height fluctuation range gradually narrowed as the A value increased, as shown in Figure 6d–o. At an A value of 2 mm, *S_z_* decreased to 3461 μm and *S_a_* to 650 μm. At an A value of 4 mm, the surface height distribution was the most uniform, with *S_z_* and *S_a_* of 3036 μm and 613 μm, respectively, representing reductions of 16.96% and 9.59% compared with the non-oscillated condition.

The scanning data show that both surface roughness and maximum surface height gradually decreased as the A value increased. Torch oscillation effectively broadened the arc heat action range and alleviated local heat concentration, making the temperature field of the thin plate more uniform and reducing residual deformation caused by uneven thermal expansion and contraction during welding. In addition, the oscillating arc continuously stirred the molten pool, promoting uniform spreading and stable solidification of the deposited metal, which improved weld formation uniformity and further reduced surface height fluctuations. These effects ultimately led to reduced surface roughness and effectively suppressed overall deformation.

### 3.3. Microstructure and Crystallographic Texture

**Figure 8:**
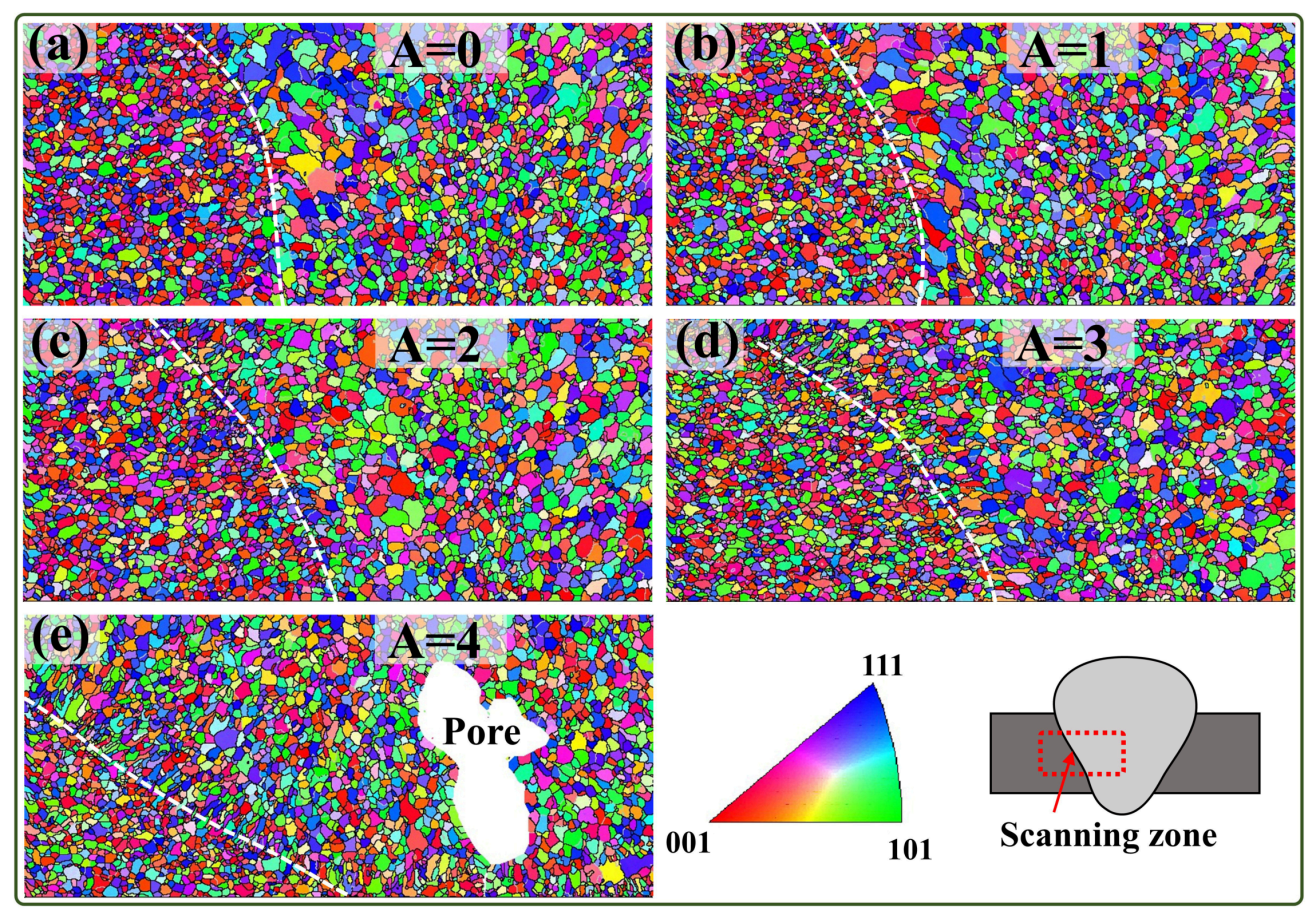
EBSD analysis of the weld region under different A values: (**a**) 0 mm, (**b**) 1 mm, (**c**) 2 mm, (**d**) 3 mm, (**e**) 4 mm. The white dashed line indicates the fusion line.

Figure 8 shows the EBSD inverse pole figure (IPF) orientation maps of the weld center zone and the region near the fusion line with and without torch oscillation. Different colors represent different crystallographic orientations of grains, and the triangle at the bottom right is the standard IPF color key. The scanning region was located on the weld cross-section. For the non-oscillated specimen, the grains in the weld center zone and the inner side of the fusion line were relatively large, with obvious coarse columnar grains oriented along the heat flow direction, and large connected regions of the same color were observed. When the oscillation amplitude increased to 1 mm, the grains began to refine, the columnar grains became shorter, and the proportion of equiaxed grains increased. The color distribution became more disordered than that at A = 0 mm, which may indicate that molten-pool stirring began to influence solidification. Molten-pool stirring may potentially induce dendrite fragmentation to produce additional potential nucleation sites. When the oscillation amplitude increased to 2 mm and 3 mm, the grains were further refined, equiaxed grains became dominant, and columnar grains almost disappeared. The grain orientation distribution became more random, and the enhanced stirring may reduce the molten-pool temperature gradient, as inferred from microstructural observations [20,30], significantly suppressing the directional growth of columnar grains. At an oscillation amplitude of 4 mm, the grains were the finest and most uniform, but obvious pore defects appeared in the map. The pores were irregular in shape and relatively large, indicating that an excessive oscillation amplitude, although providing the best grain refinement, may cause molten pool instability and increased gas entrapment, thereby worsening pore defects.

**Figure 9:**
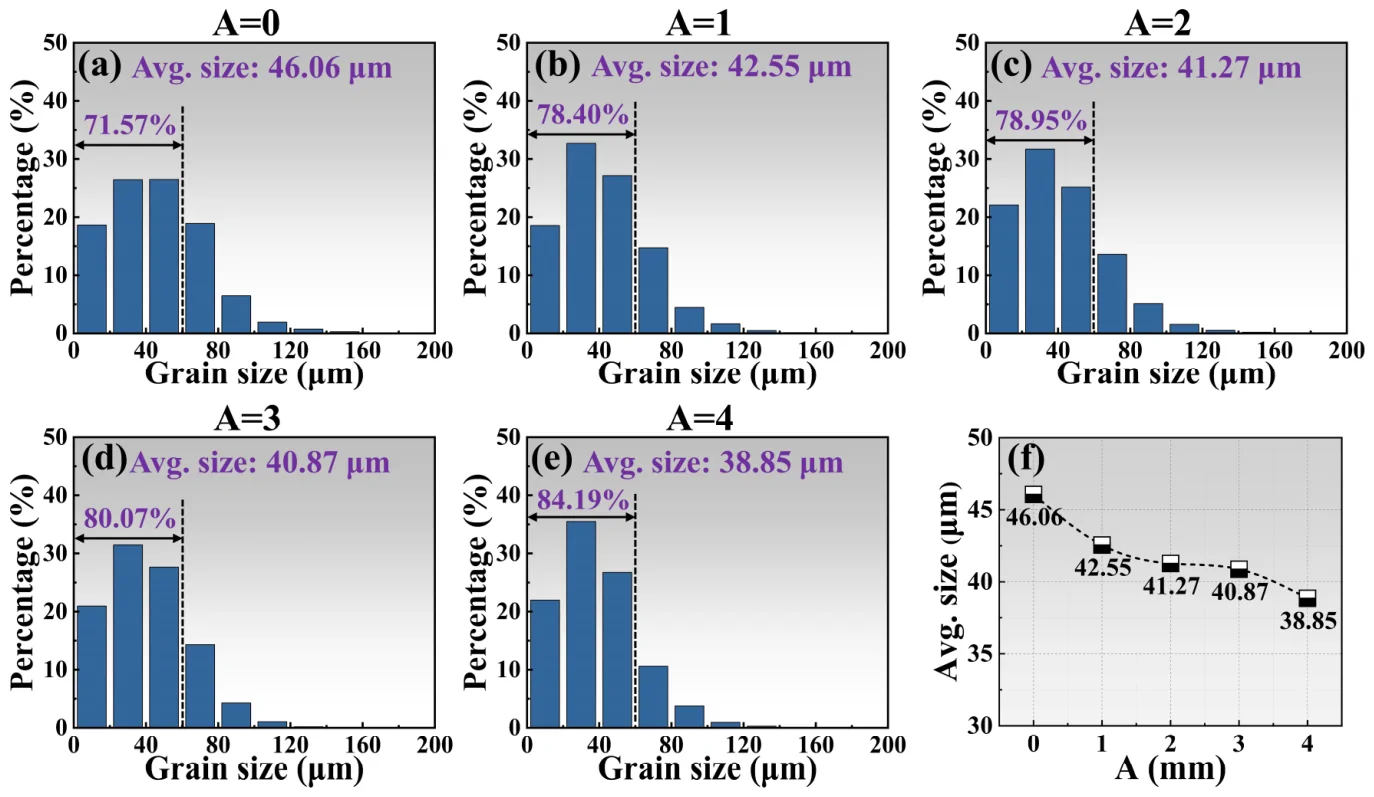
Grain size distribution and average grain size of the weld zone at different torch oscillation amplitudes: (**a**–**e**) A = 0–4 mm and (**f**) variation of average grain size with A values.

Figure 9 presents the grain size statistics for the weld center zone and the inner side of the fusion line with and without torch oscillation. At A = 0 mm (without torch oscillation), the grain size distribution was relatively wide, mainly concentrated in 20–100 μm. The average grain size was 46.06 μm and 71.57% of grains were below 60 μm. As the A value increased to 1 mm and 2 mm, the grain size distribution narrowed, the proportion of grains below 60 μm increased to 78.40% and 78.95%, respectively, and the average grain size decreased to 42.55 μm and 41.27 μm, respectively. As the A value further increased to 3 mm and 4 mm, the proportion of grains below 60 μm further increased to 80.07% and 84.19%, and the average grain size decreased to 40.87 μm and 38.85 μm. The average grain size showed a decreasing trend from 46.06 µm at A = 0 mm to 38.85 µm at A = 4 mm, a reduction of about 15.6%. However, because the EBSD analysis was based on a limited number of regions and no formal statistical test was performed, this difference should be regarded as an observed trend rather than a statistically verified difference. These results are consistent with the EBSD maps. Molten-pool stirring induced by torch oscillation may potentially trigger dendrite fragmentation to supply extra potential nucleation sites. Meanwhile, transversely redistributed heat is inferred to lower the molten-pool temperature gradient based on microstructural evidence [22,26], which suppresses columnar-grain directional growth and promotes equiaxed-grain formation. Quantitative temperature-gradient analysis is beyond the scope of the present work. Thus, the grain size decreased and the distribution narrowed as the A value increased. However, obvious pores appeared at A = 4 mm, indicating a trade-off between grain refinement and defect control at excessive A values.

**Figure 10:**
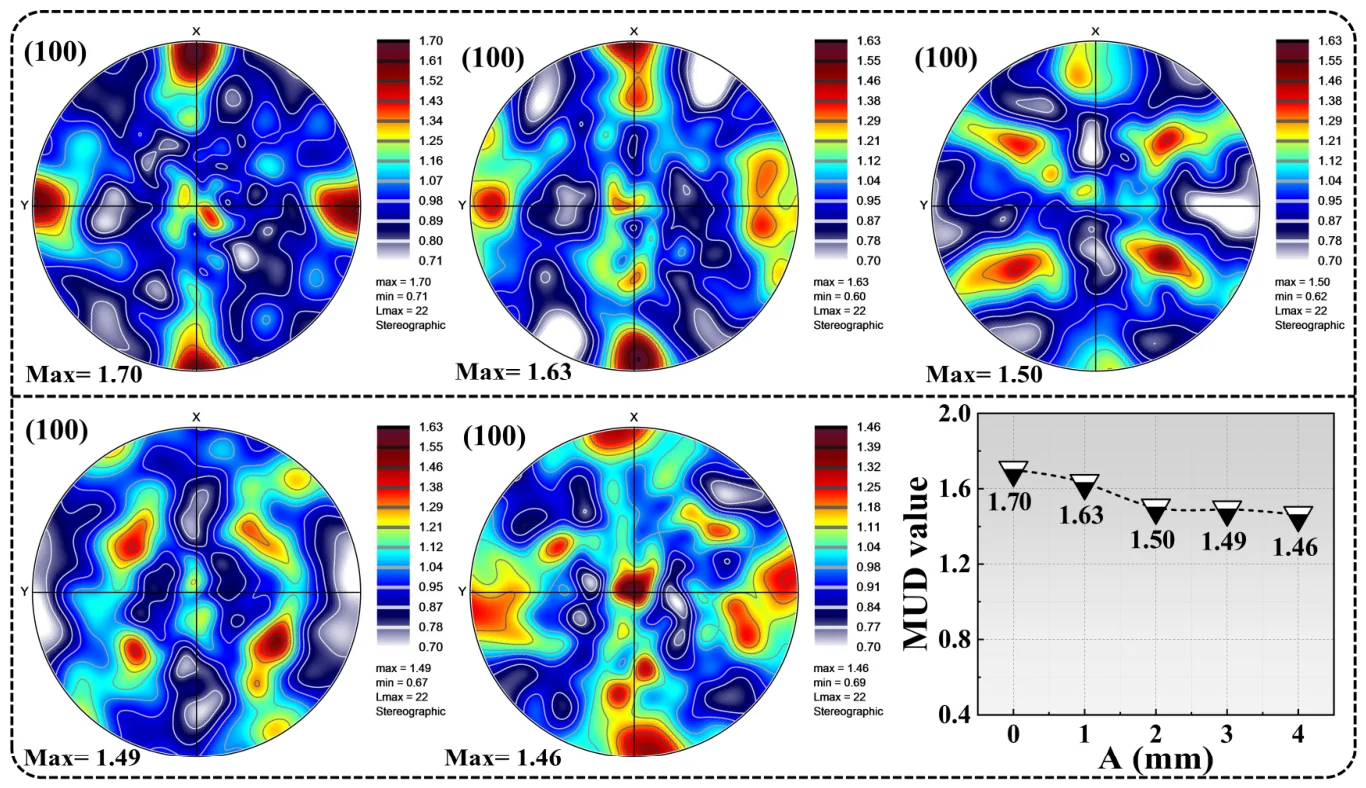
PFs and MUD values of the weld zone at different torch oscillation amplitudes.

Figure 10 shows the pole figures (PFs) of the weld zone with and without torch oscillation. Overall, the pole density remained low for all cases, and the maximum MUD values were all between 1.46 and 1.70, indicating a weak crystallographic texture and a generally scattered orientation distribution in the weld zone. At A = 0 mm (without torch oscillation), the maximum MUD was 1.70, and some color concentration regions were visible but still relatively diffuse. As the A value increased to 1 mm and 2 mm, the maximum MUD decreased to 1.63 and 1.50, and the color concentration weakened. When A was set to 3 mm and 4 mm, the maximum MUD further decreased slightly to 1.49 and 1.46, and the color distribution became uniform. The low pole density and its slight decrease with increasing A values can be explained as follows: The predominance of equiaxed grains in the weld microstructure is the fundamental reason for the low pole density values, and increasing oscillation amplitude leads to a slight decrease in the overall texture intensity.

The grain refinement and texture weakening observed in this study are generally consistent with previous arc-oscillation studies on aluminum alloys. Yan et al. reported that arc oscillation produced pronounced grain refinement in AA5052 but only limited refinement in AA6061, which was attributed to differences in crystal growth behavior and material properties rather than a change in the refinement mechanism [31]. A comparison between GTAW and SpinArc-GMAW by Umar et al. also showed that the response of AA5083 to arc manipulation depends strongly on the specific process configuration [25]. In the present study, the average grain size decreased from 46.06 μm to 38.85 μm, corresponding to a moderate grain refinement of about 15.6%, while the maximum MUD decreased only slightly from 1.70 to 1.46. This level of refinement is comparable to that reported for arc-based welding processes, where rapid solidification and a relatively steep thermal gradient limit the extent of grain refinement compared with severe plastic deformation processes such as friction stir welding. The simultaneous slight weakening of the texture is consistent with the increased fraction of equiaxed grains and the more scattered orientation distribution observed in the EBSD maps. Overall, torch oscillation in CMT welding of thin AA5083 sheets produces a moderate grain refinement and a slight texture weakening, rather than a drastic microstructural transformation.

### 3.4. Mechanical Properties and Fracture Morphology

**Figure 11:**
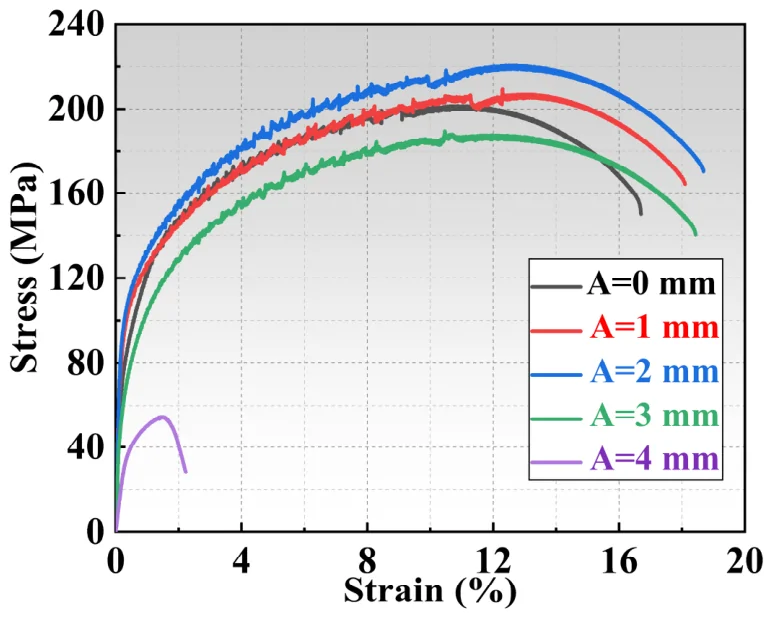
Representative tensile stress–strain curves of tensile specimens at A values ranging from 0 to 4 mm.

**Figure 12:**
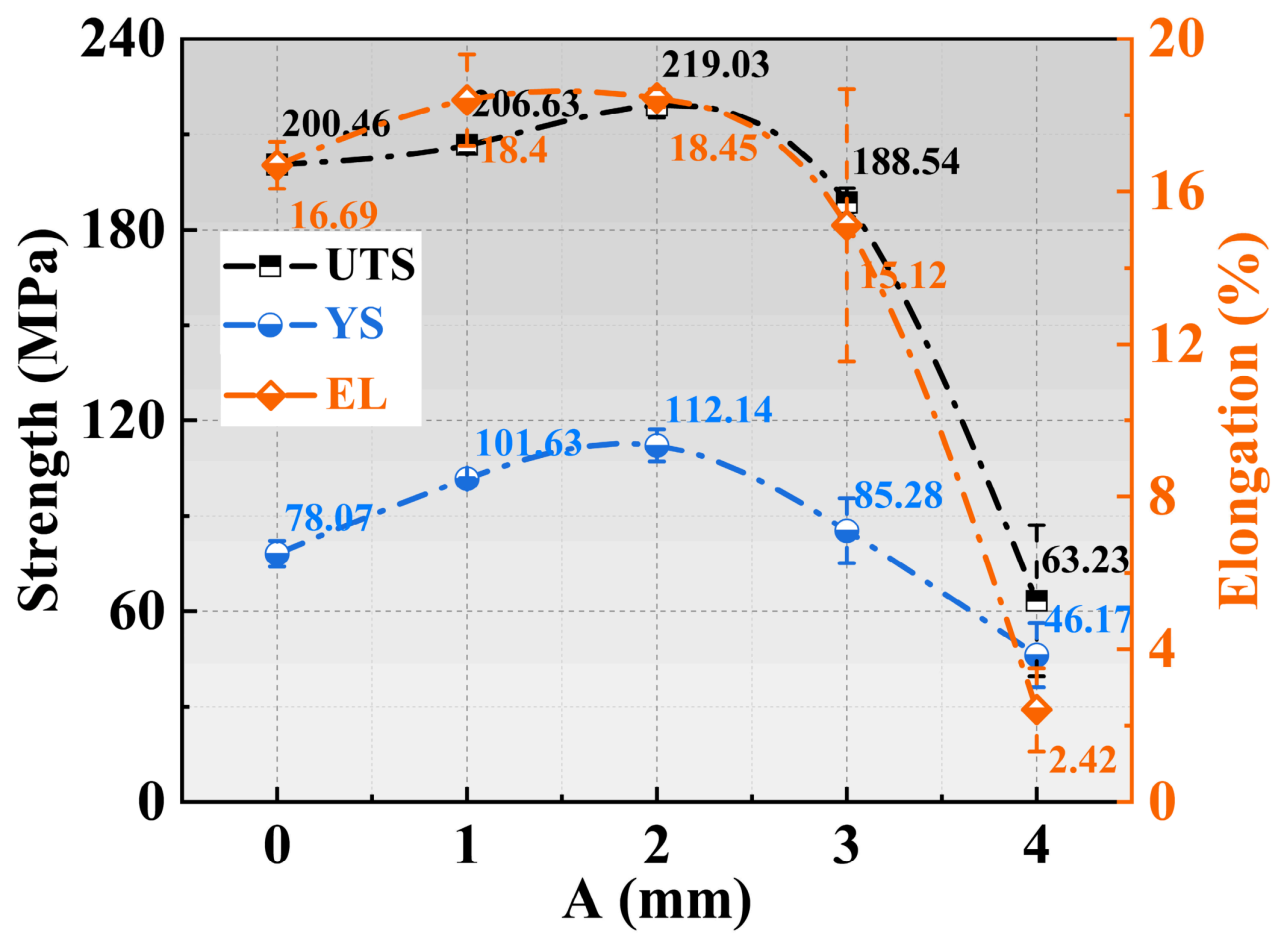
Average UTS, YS, and EL variations at different torch oscillation amplitudes.

Figure 11 and Figure 12 present the tensile curves and mechanical property statistics of butt joints with and without torch oscillation. The torch oscillation had an obvious regulatory influence on the strength and plasticity of the welded joints. Under the non-oscillated condition, the joint exhibited moderate overall mechanical properties, with ultimate tensile strength (UTS), yield strength (YS), and elongation (EL) of 200.46 ± 2.36 MPa, 78.07 ± 4.06 MPa, and 16.69 ± 0.62%, respectively, and necking and fracture occurred at a stress of about 200 MPa. When a small oscillation amplitude (A = 1) was introduced, the overall mechanical properties were effectively optimized, with UTS, YS, and EL of 206.63 ± 2.60 MPa, 101.63 ± 0.22 MPa, and 18.40 ± 1.19%, respectively. When the A value increased to 2 mm, the joint reached its optimal mechanical performance, with peak UTS increasing to 219.03 ± 3.82 MPa and YS and EL simultaneously rising to 112.14 ± 5.06 MPa and 18.45 ± 0.23%. The stress–strain curve exhibited a longer plastic deformation stage, indicating significantly improved plastic deformation capacity. As the oscillation amplitude continued to increase, the mechanical properties began to deteriorate. At an A value of 3 mm, the UTS, YS, and EL all decreased noticeably to 188.54 ± 4.52 MPa, 85.28 ± 10.20 MPa, and 15.12 ± 3.56%, and the overall mechanical performance was inferior to that of the optimal parameter. When the amplitude increased to 4 mm, the joint performance dropped sharply, and all mechanical indicators decreased substantially, with UTS, YS, and EL of 63.23 ± 23.80 MPa, 46.17 ± 10.12 MPa, and 2.42 ± 1.09%, respectively. The specimen fractured at extremely low strain, and the stress–strain curve showed obvious abnormal fluctuations, demonstrating that excessive oscillation parameters introduced severe welding defects and ultimately led to joint failure. Although no formal statistical hypothesis testing was performed, the 18.57 MPa rise in average UTS exceeds the standard deviation range of both groups, indicating a measurable improvement under the present experimental conditions.

Torch oscillation significantly affects the performance of butt joints by regulating welding deformation, microstructure, and defect state. A small oscillation amplitude (A = 1–2 mm) effectively improves the overall joint performance. Under the non-oscillated condition, the arc heat input is concentrated at the weld center, and the large temperature gradient causes uneven cooling and shrinkage of the plate, resulting in obvious welding deformation. Moderate oscillation broadens the arc action range and homogenizes the welding temperature field, effectively alleviating thin-plate deformation. At an amplitude of 2 mm, the surface flatness of the base plate is significantly improved, and the tensile load distribution becomes more uniform. Meanwhile, arc oscillation refines the weld grains and promotes equiaxed grain formation, improving microstructural uniformity and plastic deformation capacity, thereby achieving the best comprehensive mechanical properties within the investigated parameter range.

When the oscillation amplitude reaches 3 mm or above, the mechanical properties deteriorate significantly. Excessive torch oscillation disturbs the stability of the molten pool and increases gas entrapment, making pores and local lack of fusion more likely to form. These defects act as crack initiation sites and propagate rapidly under tensile loading, severely degrading the performance of the butt joint. The A = 4 mm specimen fractured at low strain with an elongation of only 2.42%, representing a typical defect-induced failure mode. In summary, an optimal process window exists for torch oscillation. An amplitude of 2 mm simultaneously achieves deformation suppression, grain refinement, and controllable defects, yielding the best mechanical properties. In contrast, the negative effects of defects caused by excessive oscillation offset the benefits of microstructural and deformation optimization, ultimately leading to a sharp decline in joint performance.

**Figure 13:**
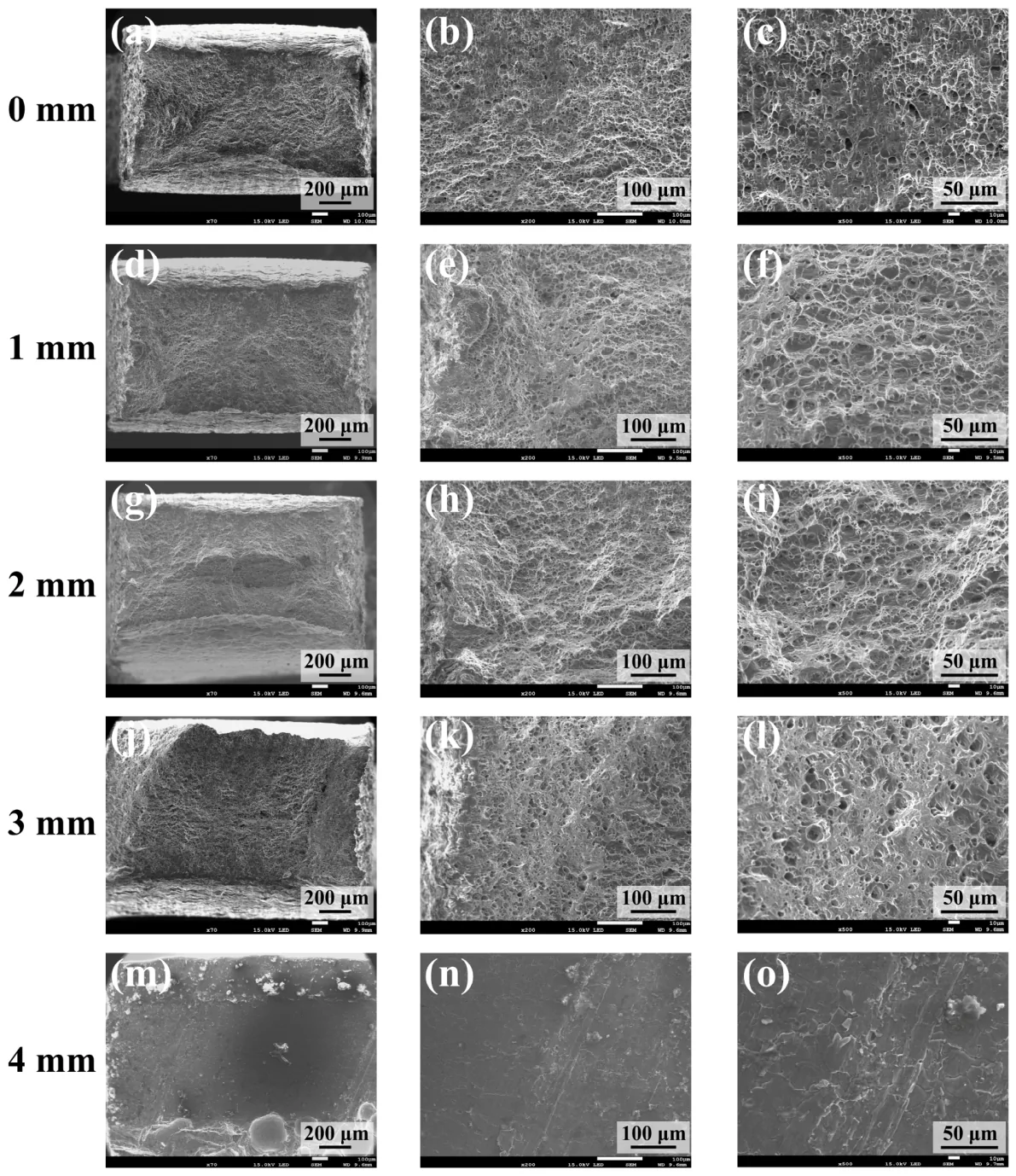
Fracture morphologies of tensile specimens from butt joints at different torch oscillation amplitudes: (**a**–**c**) 0 mm, (**d**–**f**) 1 mm, (**g**–**i**) 2 mm, (**j**–**l**) 3 mm, and (**m**–**o**) 4 mm.

Figure 13 shows the fracture morphologies of tensile specimens from 5083 aluminum alloy CMT butt joints at different torch oscillation amplitudes. The first column presents low-magnification macroscopic fractures, and the second and third columns present local high-magnification images. For the non-oscillated control group (A = 0 mm), the macroscopic necking was insignificant, and the fibrous zone was relatively small. In the high-magnification images, the dimples varied in size and depth, and flat regions were locally present, indicating insufficient plastic deformation before fracture. At A = 1 mm and A = 2 mm, the ductile fracture characteristics were markedly enhanced, with more pronounced macroscopic necking, an enlarged fibrous zone, and more numerous dimples of uniform size. The A = 2 mm specimen exhibited the most prominent necking and the densest and deepest dimples, indicating the best ductility, which is consistent with the peak tensile properties of this group. At A = 3 mm, the macroscopic necking weakened, the number of dimples decreased, the dimple size became uneven, and cleavage steps appeared locally, indicating reduced ductility. At A = 4 mm, almost no necking was observed, and the fracture surface was flat. At high magnification, dimples were rarely observed. Cleavage-like facets and river-like patterns can be seen; these features arise from rapid crack propagation triggered by pre-existing pores and lack-of-fusion defects rather than intrinsic cleavage of defect-free weld metal. Obvious crack-initiation sites and secondary cracks are visible, corresponding to premature brittle-mode failure driven by welding defects. The pores and lack-of-fusion defects caused by excessive oscillation amplitude acted as crack initiation sites, leading to brittle fracture of the joint at low strain. The evolution of fracture morphology is highly consistent with the variation in tensile mechanical properties.

Fracture mode evolves gradually with increasing torch-oscillation amplitude, covering ductile, mixed ductile-brittle, and defect-driven brittle-mode failure. For the non-oscillated condition (A = 0 mm), the fracture belongs to a predominantly ductile mode with uneven dimples. Moderate welding residual deformation produces local stress concentration during tension; heterogeneous plastic deformation occurs via dislocation motion, and voids nucleate at second-phase particles, then grow and coalesce to form dimples. Limited necking indicates that residual stress slightly suppresses uniform plastic deformation. At A = 1 mm and A = 2 mm, typical ductile fracture dominates. The refined, more homogeneous weld microstructure facilitates uniform dislocation slip and delays local strain concentration. Voids nucleate uniformly across the fracture plane, producing dense, deep, and evenly distributed dimples and obvious macroscopic necking. The reduced welding-induced residual deformation further homogenizes tensile stress distribution. This microstructural improvement, together with lowered residual stress, contributes to the highest ductility and strength within our tested parameter window. When amplitude rises to A = 3 mm, mixed ductile-brittle fracture occurs. Small pores begin to form inside the weld. Under tensile loading, these pores act as initial crack sources. Voids still coalesce to form dimples in local regions, while crack propagation originating from pre-existing pores generates cleavage-like facets. The coexistence of dimple regions and brittle-like facets corresponds to degraded strength and elongation. At A = 4 mm, abundant pores and local lack-of-fusion defects are distributed in the weld zone. These large-size welding flaws become preferential crack-initiation sites. Cracks rapidly propagate along defect interfaces under low applied strain. Dislocation-mediated plastic deformation is largely suppressed. Consequently, nearly no necking can be observed, and failure occurs in a defect-driven brittle mode, even though the weld matrix itself possesses a ductile Al-Mg microstructure.

Overall, torch oscillation alters failure behavior via two competing pathways: (i) moderate oscillation refines grains and mitigates welding deformation, which promotes homogeneous dislocation activity and improves ductility; (ii) excessive oscillation destabilizes the molten pool, introduces pores and lack-of-fusion defects, accelerates crack propagation, and triggers premature failure. The balance between these two competing effects determines the final tensile performance and fracture mode.

## 4. Conclusions

CMT butt welding of 2 mm-thick AA5083-H112 plates was performed using ER5183 welding wire. The influences of torch oscillation on weld geometry, plate deformation, microstructure, and mechanical properties were systematically studied. The main conclusions are summarized below:

(1) Torch oscillation redistributes the arc heat input, so a larger A value increases the weld width while decreasing the depth of penetration. At A = 4 mm, a complete lack of penetration occurs together with large circular pores. The weld reinforcement remains stable due to the mutual offset of two competing effects.
(2) Torch oscillation alleviates the out-of-plane deformation of the thin plates, and both *S_z_* and *S_a_* decrease as the A value rises. When the A value increases to 4 mm, *S_z_* and *S_a_* are reduced by 16.96% and 9.59%, respectively. The homogenized temperature field and molten-pool stirring jointly improve the surface state.
(3) The oscillating arc refines the weld grains, and the overall texture intensity decreases slightly with rising oscillation amplitude. Dendrite fragmentation, together with a reduced temperature gradient, may contribute to equiaxed-grain formation. The average grain size decreases from 46.06 μm at A = 0 mm to 38.85 μm at A = 4 mm, and no obvious texture forms in any of the welds. However, severe pores appear at 4 mm despite the finest grains.
(4) The butt joint achieves the highest performance at A = 2 mm among the investigated amplitudes under the present welding conditions, and UTS, YS, and elongation reach 219.03 MPa, 112.14 MPa, and 18.45%, owing to suppressed deformation, grain refinement, and an acceptable defect level. Higher A values (≥3 mm) introduce pores and lack of fusion, causing brittle fracture and degraded performance, consistent with the fracture morphology.
(5) An effective process window exists for torch-oscillation CMT welding of 5083 thin plates, in which 2-mm torch oscillation realizes a good balance among deformation control, grain refinement, and defect suppression for the welding parameters adopted in this study. Excessive oscillation produces detrimental defects that override the microstructure-related benefits.

## Figures and Tables

**Table 1:** Compositions of base plates and welding wire (wt.%).

Materials	Mg	Mn	Cr	Ti	Cu	Zn	Fe	Si	Al
AA5083	4.58	0.64	0.16	0.03	0.05	0.25	0.31	0.29	Bal.
ER5183	4.91	0.63	0.08	0.10	0.04	0.01	0.10	0.03	Bal.

**Table 2:** Welding process parameters used in this study.

No.	Wire Feed Rate (m/min)	Welding Speed (m/min)	Welding Current (A)	Arc Voltage (V)	Oscillation Frequency (Hz)	A (mm)
1	5.2	0.4	70	12.2	5	0
2						1
3						2
4						3
5						4

## Data Availability

The original contributions presented in this study are included in the article. Further inquiries can be directed to the corresponding authors.
